# Undereporting of acute pesticide poisoning in Tanzania: modelling results from two cross-sectional studies

**DOI:** 10.1186/s12940-016-0203-3

**Published:** 2016-11-29

**Authors:** Elikana E. Lekei, Aiwerasia V. Ngowi, Leslie London

**Affiliations:** 1 Tropical Pesticides Research Institute, P.O.Box 3024, Arusha, Tanzania; 2 Department of Environmental and Occupational Health, Muhimbili University of Health and Allied Sciences (MUHAS), School of Public Health and Social Sciences, P O Box 65015, Dar es Salaam, Tanzania; 3 School of Public Health & Family Medicine - Faculty of Health Sciences, Anzio Road, Observatory, 7925 Cape Town, South Africa

**Keywords:** Acute pesticide poisoning, Underreporting, Modeling, Tanzania

## Abstract

**Background:**

Acute pesticide poisoning (APP) is known to cause serious injuries to end users globally but the magnitude of this problem in Tanzania is not well known. This study aimed to determine the extent and pattern of underreporting of APP in Tanzania to inform the development of a surveillance system and appropriate interventions.

**Methods:**

This study integrates findings from two recent Tanzanian studies. A household survey established the proportion of poisoned farmers in a typical rural area who reported to hospital for a pesticide poisoning. Only 5 of the 112 farmers who reported attending hospital due to poisonings could be traced in medical records at the facilities they claimed to have attended. The 95% confidence interval for this ratio (5/112) was used to generate a high and low boundary for the estimates. Three under-estimation factors were generated for sensitivity analysis to adjust for under-reporting. A review of health facilities in three regions of Tanzania collected prospective data on admissions for APP in 2006 to generate population-based APP incidence rates stratified by circumstances of poisoning (occupational, accidental, suicide, and unknown). Sensitivity analysis was conducted involving adjustment for high and low boundaries of the under-reporting of occupational APP and an adjustment for different scenario allocations of cases with ‘unknown’ circumstances to different combinations of known circumstances.

**Results:**

The study estimated the rate of occupational poisoning as ranging from 11.3–37.7 cases/million to 84.3–279.9 cases per million. The rate of all poisonings (occupational and non-occupational) ranged from 24.45–48.01 cases per million to 97.37–290.29 cases per million. Depending on the choice of scenario and under-reporting correction factor used, occupational APP could comprise from 52.2 to 96% of all APP cases.

**Conclusion:**

The study confirms that data on APP in Tanzanian hospitals are poorly reported and that occupational circumstances are particularly overlooked in routine facility-based surveillance. Occupational APP needs to be taken more seriously in addressing prevention measures. A comprehensive surveillance system for APP should consider multiple data sources including community self-reporting in order to achieve better coverage.

## Background

Acute pesticide poisoning (APP) is recognised as a major cause of injury for farmers and users of pesticides globally but the magnitude of the problem in Tanzania is not well known. The yearly worldwide prevalence of APP has been estimated at about 1,000,000 unintentional and 2,000,000 intentional cases, with approximately 220,000 deaths per year [1, 2]. Studies in different parts of the world report incidence rates of acute pesticide poisoning of 20/100,000 in Central America [1], 2300/100,000 in Nicaragua [3], 180 per 100,000 in Sri Lanka [4] and 4.2/100,000 in South Africa [5]. These differences in rates reflect differences in use conditions, toxicity of agents and, most likely, sources used to compile the data [6].

Nonetheless, these figures are consistent with reports of the problematic hygiene and safety conditions under which these products are used in many developing countries [7–15]. However, it is likely that these statistics underestimate the real burden caused by pesticides [16]. A study conducted in Nicaragua in 1996 to estimate the rate of underreporting of pesticide poisoning cases to a poisoning registry found that 65% of poisoning cases were not reported [17]. Underreporting of pesticide poisoning data in a national notification database has also been reported to be of the order of 90% in South Africa [5]. Most published studies rely on data from cases presenting to health facilities and recorded in national registries or health information systems and therefore include only the most obvious poisonings such as those requiring hospitalization. This means that the real toll from pesticides, including milder and non-hospitalised cases, is likely to be greater than reported. Some symptoms due to pesticides poisoning are very non-specific and may be easily confused with other diagnoses, particularly if health care providers are not well trained [18, 19]. Farmers who suffer mild poisoning may not regard the symptoms as sufficiently important to seek health care, or may have become habituated to symptoms as ‘normal’ for farming practice [20]. Those suffering acute poisoning or high episodes of exposure might also end up with long term effects [21]. Therefore, despite the apparent magnitude of this problem, accurate data to inform public health decision-making about the risks of pesticide poisoning is lacking.

Tanzania has a well-established Health Management Information System (HMIS) collecting routine data from all health facilities in Tanzania through the Ministry of Health. The HMIS came into operation across Tanzania in 1997 and collects out-patient data, in patient demographics,type of visit, diagnosis, treatment and referrals, as well as admission data through a register on ward, name, address, next-of-kin, age, diagnosis, date discharged and final outcome (death, recovery or referral). The districts receive raw data from health facility reports and transfer the data immediately to a district file, which is then transmitted to the regions and finally to the Ministry of Health. The HMIS covers a range of health care levels, including community health at the village level, health care centers, district hospitals, regional and referral hospitals [22]. Information flows through the health care system to and fro including notifications for notifiable diseases such as cholera and meningitis. Among the weaknesses of the HMIS are incomplete data collection resulting in missing data, weak data presentation, poor data accessibility, high workload of health staff and poor availability of processed information when needed [23]. In the HMIS, APP falls into a category known as “Poisoning” including all poisoning cases arising from pesticides, kerosene, drugs, snake bites, insect bites plants and other agents. APP therefore does not have its own specific diagnosis category in the register. Identifying APP therefore requires active extraction of data from facility records, using the register to identify cases.

This study aimed to determine the extent and pattern of underreporting of APP in Tanzania for better estimation of the real rate of APP in order to inform the development of a surveillance system and implement appropriate interventions to reduce the burden from APP.

This study is based on a larger project investigating surveillance for APP in Tanzania [24] and integrates findings obtained from two studies conducted in Tanzania by the same authors [20, 24] for the purpose of modelling more accurately the rates of acute pesticides poisoning in Tanzania. The two studies were conducted at the same time period and the methods for these studies are briefly outlined below.

A household survey of 121 farmer heads of households from Arumeru district in the Arusha region, using a standardized questionnaire documented previous histories of APP (self-reported), health-seeking behavior related to the APP and general safety and hazard knowledge and storage practices [20]. Unsafe pesticide handling practices were assessed through observation of pesticide storage, conditions of personal protective equipment (PPE) and through self-reports of pesticide disposal and equipment calibration. This study established the proportion of farmers reporting APP, the proportion of these poisoned farmers who sought health care at a health facility and the proportion of farmers seeking health care for whom hospital records could confirm the poisoning.

A second study reviewed hospital admission data for APP retrospectively (2000–2005) in 30 facilities in four regions of Tanzania and collected prospective APP data over 12 months in 2006 focused on 10 facilities with the highest reporting of APP in the retrospective study. The study was conducted in four coffee and vegetable agricultural areas of Tanzania namely Mwanza, Iringa, Kilimanjaro and Arusha [24].

This study compared prospective to retrospective data collection under the assumption that prospective data collection, which was supported by intensive training and awareness raising amongst providers, was less likely to miss cases of APP and was more likely to generate rates closer to the underlying rate of APP for the facility drainage regions concerned. This was born out by lower proportions of cases with missing data on prospective data collection (for example, providers were more likely to record circumstances of poisoning and the agent involved, and register pages were less like to be lost) [24]. For this reason, the prospective cohort data were therefore used to derive an APP incidence rate for 2006. The rate was estimated for the three regions concerned, with a profile generated of the relative contributions of different circumstances (suicide, accidental, occupational, homicide and unknown) to the overall number of cases.

These two studies provided the main input data for this study, which was to determine the extent and pattern of underreporting of APP in Tanzania.

## Methods

### Estimating the burden of APP

Estimating the burden of APP, accounting for under-reporting of occupational APP involved four steps as outlined in Table 1.

**Table 1:** Overview of the method

Step 1	Health facility admissions in 3 regions of Tanzania over the period of 12 months were monitored prospectively from Jan 2006 to December 2006 using intensified surveillance to generate population-based estimates for the incidence of APP, stratified by circumstances of poisoning (occupational, accidental, suicide, homicide and unknown circumstances). These generated baseline APP rates for further adjustment.
Step 2	Data from a household survey of farmers was compared to records in health facilities to generate a ratio of occupational APP cases reported at health facilities, reflecting the extent of under-reporting of APP cases due to occupational circumstances. The 95% confidence interval for this ratio was used to generate a high and low boundary for the estimate.
Step 3	Cases of APP in the prospective study for which circumstances were unknown were allocated in a sensitivity analysis to each of the other four known circumstances^a^, and the contribution of newly allocated occupational APP adjusted for under-reporting.
Step 4	The rate of occupational APP recorded at health facilities was adjusted to account for the under-reporting of occupational APP identified in Step 2, including high and low boundary estimates, and to account for reallocation of unknown to known circumstances as identified in in Step 3.
Step 5	Total APP incidence rates, summing across all circumstances, were generated under the different contingencies identified. The contribution of occupational poisoning as a proportion to overall APP incidence was described.

^a^ Known circumstances included suicide, occupational, accidental and homicide. However, because cases with homicide were so few (1.1% of all cases), the sensitivity analysis included homicide within the category accidental for allocation and only three circumstances were used for allocation: suicide, accidental and occupational

This process generated an overall estimate of the underlying rate of APP in the study area. Two sensitivity analyses were included in the process – firstly, an adjustment for high and low boundaries of the under-reporting of occupational APP; and an adjustment for different allocations of cases with ‘unknown’ circumstances to different combinations of known circumstances.

### Baseline rates of APP in 3 regions in Tanzania

Prospective data collection at 10 facilities in three regions in Tanzania using a standardised data collection tool generated a total of 230 cases from January to December 2006. The definition of an APP used in this study was a diagnosis of APP made by the clinician attending the patient and recorded in the register, patient folder or both. In general, the diagnosis was based on a history of exposure (from the patient, relative or accompanying person) to one or more pesticides and clinical manifestations of poisoning or specific laboratory test results compatible with poisoning, within 14 days of exposure. The 10 facilities were included on the basis of reporting high numbers of APP in a preceding retrospective review [24] and staff were intensively trained to record cases of APP. Prospective data collection improved the quality of data collected, reducing the amount of missing data for age (16%), poisoning agent (16%), outcome (39%) and circumstance of poisoning (57%) [18]. The definition for a case of APP was a person who, after any exposure to one or more pesticides, presented clinical manifestations of poisoning within 14 days of exposure or presented a medical history of having been poisoned or whose relative / accompanying person gave a history of the patient having been poisoned. Data were collected on how the exposure happened, the circumstances of exposure, the agent responsible for poisoning and other relevant details. However, absence of such data did not exclude the case. The primary inclusion criterion was a diagnosis of APP made by the clinician attending the patients and recorded in either the register, patient folder or both.

Denominator data for incidence, stratified by age and gender for the study regions Kilimanjaro, Mwanza and Arusha, were drawn from a national census conducted in 2002 by the Tanzania Bureau of Statistics which was adjusted for annual population growth of 2.091% to reflect population estimates for 2006. Rates were estimated according to four circumstances (occupational, accidental, suicide and homicide). Because of small number of homicide cases (*n* = 1), homicide and accidental were combined in one category. Where data were not available on circumstances, this was coded as ‘unknown’ circumstances.

### Estimating under-reporting of occupational APP

In the household survey [20], 92.5% (*n* = 112) of the 121 respondents reported a past APP, all of which were due to occupational circumstances. A case of previous APP as defined in study 1 was defined as any self-reported short-term illness or health effects confirmed by the researcher as consistent with exposure to the pesticide exposure reported by the respondent. This approach to capturing the full range of APP has been used in other studies in developing countries [25–31].

Of the 23 farmers who reported attending a health facility for occupational APP in the previous year, only 5 (21.5%) were traceable in local health facility records. There were an additional 89 farmers who reported experiencing a past occupational APP who did not seek health care for their poisoning. This meant that only 5 of all 112 poisoning incidents (or 4.5%) could be traced in the routine Health Information System as presenting to local facilities for an APP. Using these data to model the unreported occupational cases, which were mostly mild poisonings, suggests that the proportion of occupational poisonings reported in hospital information systems is 0.045 with a 95% CI of 0.014-0.104 (i.e. between a low of 1.4% to a high of 10.4%). The inverse of these proportions represents the factor by which APP cases with recorded occupational circumstances would need to be multiplied in order to obtain more accurately the number of occupational APP in the study area (called an ‘under-estimation factor’). For the three estimates, the underestimation factors derived were therefore (a) for the lower margin of the 95% confidence interval, 1/0.014 = 71.4; (b) for the point estimate, 1/0.045 = 22.2; and (c) for higher margin, of the 95% confidence interval, 1/0.104 = 9.6.

Approximately 19% of the 230 cases recorded prospectively in the hospital reporting system were APP cases with unknown circumstances (*n* = 44) [24]. Redistributing this group of APP cases with unknown circumstances across different categories of known circumstances was conducted in a sensitivity analysis with different assumptions for the redistribution:

- All cases with unknown circumstances allocated to suicide;
- All cases with unknown circumstances allocated to accidents/homicide;
- All cases with unknown circumstances allocated to occupational circumstances;
- All cases with unknown circumstances distributed equally among suicide, occupational or accidents/homicide; and
- All cases with unknown circumstances distributed proportionally to their existing baseline distribution for known cases (in proportion to baseline rates for suicide, occupational and accident/homicide circumstances of 6.71: 1.18: 3.67).

The total number of APP cases (*n* = 230) was kept constant in this reallocation. Options (a) and (b) above make no impact on the number of occupational APP cases, so for the purposes of the Sensitivity Analysis below, are treated as the same contingency. There are thus four possibilities in adjusting occupational APP for unknown circumstances: (a)/(b) in which no additional cases are allocated to Occupational APP; (c) in which all 44 cases with unknown circumstances allocated to occupational circumstances; (d) in which the 44 cases with unknown circumstances are distributed equally amongst the different circumstances and (e) in which the 44 cases with unknown circumstances are distributed in proportion to the baseline distribution of the different circumstances.

To estimate more accurately the number of cases involving occupational circumstances from hospital-reported APP [24], the rate for occupational APP obtained above was multiplied by the underestimation factor derived from the farmer head-of-household survey., in this case 1/0.045 = 22.2; for the lower margin of the 95% confidence interval; the factor used was 1/0.014 = 71.4 and for higher margin, the factor was 1/0.104 = 9.6. Thus three under-estimation factors were generated for sensitivity analysis to adjust for under-reporting – a point estimate of 22.2, a high estimate of 71.4 and a low estimate of 9.6.

Total APP incidence rates, summing across all circumstances, were generated under the different contingencies identified accounting for underestimation of occupational APP. The total APP incidence increase depended on the under-estimation factor used. Further, the reallocation of unknown circumstances did not directly change the total APP incidence rate but depending on whether a particular reallocation added to the number of occupational APP cases, the underestimation factor applied to this amended occupational APP estimate then multiplied out to a further increase in total cases, and therefore to an increase in total APP incidence. The contribution of occupational poisoning as a proportion to overall APP incidence under the different assumptions was then described.

## Results

Of the total 230 cases of APP recorded over the year, 8% were recorded as occupational circumstances and 19% were of unknown circumstances (Table 2).

**Table 2:** Sensitivity analysis of APP rates^a^ per 1000000 by circumstances under different redistribution allocations for ‘unknown’ circumstances

Circumstance	n	%	Baseline rate per 1,000,000	APP Rates per 1,000,000 under different scenarios				
	Circumstance data table	% by circumstance	No Redistribution	(a) all unknown to Suicide	(b) all unknown to Accident/Homicide	(c) all unknown to Occupational	(d) unknown redistributed equally^b^ to other categories	(e) unknown allocated proportionally^c^ to other categories
(a) Unknown	44	19.13	2.74	0.00	0.00	0.00	0.00	0.00
(b) Suicide	108	46.96	6.71	9.45	6.71	6.71	7.63	8.30
(c) Accidental/ homicide	59	25.65	3.67	3.67	6.41	3.67	4.58	4.54
(d) Occupational	19	8.26	1.18	1.18	1.18	3.92	2.09	1.46
Total	230	100.00	14.30	14.30	14.30	14.30	14.30	14.30
Sum of circumstances other than occupational (a + b + c)			13.12	13.12	13.12	10.38	12.21	12.84

^a^ The baseline rates were obtained in a prospective follow-up of 10 facilities in four regions of Tanzania over 12 months in 2006

^b^The 44 cases of APP were allocated in equal proportion to suicide, occupational circumstances and accident/homicide

^c^The 44 cases of APP with were allocated in proportion to the baseline rates of APP for suicide, occupational circumstances and accident/homicide (6.71: 1.18: 3.67, respectively)

Modelling more accurately the rate of occupational APP involved two sets of contingencies – adjusting for occupational cases whose circumstances were missed and appeared as ‘unknown’ and for under reporting of occupational APP in routine health information systems. Table 2 presents the sensitivity analysis for reallocating unknown circumstances to occupational circumstances.

The sum of the APP rates for circumstances other than occupational is only slightly affected by the different adjustments. With no allocation to occupational circumstances (baseline and options (a) and (b)), there is no change in the rate of APP due to non-occupational circumstances (13.12 per 1,000,000). In the scenarios where different proportions of APP with unknown circumstances are allocated to occupational APP, the sum of the APP rates for circumstances other than occupational varies from 10.38 (option c) to 12.84 per 1000 000 (option (e) (Table 2). The rates for APP due to occupational circumstance vary only in options (c) to (e), with the highest being a rate of 3.92 per 1,000,000, an increase of over 3-fold for occupational circumstances.

The second set of contingencies examined in the modelling involves adjusting for under-reporting of occupational APP in routine facility-based health information systems. Table 3 models the rate of occupational APP adjusting in the three right-hand columns the different proportions allocated to occupational APP (options c, d and e, from Table 2 above), and the rows applying the high, median and low correction factors for underestimation (71.4, 22.2 and 9.6, respectively) to include occupational APP cases occurring in the community that are not detected from routine facility-based surveillance.

**Table 3:** Estimates for Occupational APP Incidence Rates (Cases/1000000 – results of sensitivity analyses

Under-estimation Correction Factor	IRs derived from the different scenario allocations to occupational poisoning			
	Scenario (a) and (b) 1.18	Scenario (c) 3.92	Scenario (d) 2.09	Scenario (e) 1.46
9.6	11.328	37.632	20.064	14.016
22.2	26.196	87.024	46.398	32.412
71.4	84.252	279.888	149.226	104.244

The underlying rate of APP due to occupational circumstances, including both facility-reported and APP in the community, for the study sites based on the lower boundary of the correction factor for under-estimation would therefore be between 11.3 to 37.6 cases/1,000,000 (Tables 2 and 3) whereas based on the upper boundary of the correction factor, rises to a maximum of 84.3 to 280.0 cases/1,000, 000 (Table 3).

Combining occupational and non-occupational APP cases under the different allocation scenarios for unknown circumstances, and under the different correction factors for occupational APP, the total APP rate due to occupational and non-occupational circumstances, based on the lower margin of the 95% CI for under-reporting of community-based APP ranged from 2.45 to 4.80 cases/1000000 for the lower boundary of the correction factor. Based on the upper boundary of the correction factor, the underlying rate of APP including both occupational and non-occupational circumstances ranged from 9.74 to 29.03 cases/1,000,000 (Table 4).

**Table 4:** Summation of occupational and non-occupational IR for APP

Underestimation factor	Scenario	IR for APP (Cases/million) in occupational, non-occupational and all circumstances)			
		Scenario (a) and (b)	Scenario (c)	Scenario (d)	Scenario (e)
	Non occupational^a^	13.12	10.38	12.21	12.84
9.6	Occupational	11.33	37.63	20.06	14.02
	Total	24.45	48.01	32.27	26.86
22.2	Occupational	26.19	87.02	46.39	32.41
	Total	39.31	97.40	58.60	45.25
71.4	Occupational	84.25	279.89	149.23	104.24
	Total	97.37	290.27	161.44	117.08

^a^ Non-occupational APP does not change within each scenario– only the under-estimation of occupational APP changes depending on the under-estimation factor used

The modelling suggests that occupational circumstances are a substantially higher proportion than reported in baseline (8% - Table 2). Depending on which scenario and which under-reporting correction factor is used (Table 4), occupational APP could comprise from 52.2 to 96% of all APP cases.

## Discussion

The findings of this study suggest that both APP due to occupational causes and APP in general are substantially underestimated in routine facility-based surveillance systems. The contribution of occupational APP, when including cases that do not present to health facilities, can comprise between 50 and 96% of all APP cases. These are cases reported by farmers sustaining APP in the community who do not seek health care, for one or other reason. Data from other Tanzanian [20] suggest that farmers do not see health facilities as accessible or suitable for dealing with these kinds of poisonings. As a result, data from routine health facility surveillance can miss important contributions to morbidity arising from pesticide exposure in a developing country setting like Tanzania.

The implication of this underreporting are important in that the real burden arising from APP is not evident to key decision makers and, as a result, the Government is not pressed to take action for intervention measures.

The modelled IR for APP found in this study (ranging from 3.7 cases/1000 000 to 27.9 cases per 1000 000 is lower than rates reported in Nicaragua (23 000/1,000,000) [3], Bolivia (780/1,000,000) [32] and Sri Lanka (3180/1000000) [33] but close to rates reported in Central America (20/1000000) [34]. The differences could be a result of higher exposure circumstances in parts of Latin America and perhaps greater toxicity of the products handled compared to Tanzania which has very few Class I agents, all of which are restricted. Alternatively, differences could also arise, as explained earlier, from the different methods used for data collection, with self-reported data used in the study by Corriols et al., 2009 [3]. However, at the very least, broadly speaking, it is clear there is better characterization of APP using the data in this study that generates incidence rates for APP in Tanzania that are of the same order as that obtained in studies done in other Lower-Income countries.

What the study also highlights is the importance of a comprehensive surveillance system for APP. Effective surveillance needs to rely on data from multiple sources since surveillance at health care facilities tend to document severe poisonings and miss a substantial number of cases. Community self-reporting is a potential complementary data source and has presented promising results in studies conducted in Tanzania. The Tanzanian NGO’s namely AGENDA and TAPOHE utilized the FAO self-reporting package from East Asia and worked with the Tropical Pesticides Research Institute (TPRI) in a small farming community in Arumeru and Karatu districts in Arusha, and in Mgeta and Turiani districts in Morogoro to pilot community self-reporting of APP, which was also being undertaken by Work and Health in Southern Africa (WAHSA) at Ngarenanyuki ward in Arusha. The Ministry of Agriculture, Food Security and Cooperatives (MAFS) started community self-reported APP studies in Kilolo district in Iringa region (which has extensive vegetable cultivation) as part of its PIC commitments. These pilots appear to have worked successfully to capture data [35–37] particularly non-severe poisoning cases. However, harmonization of these different surveillance activities will be needed in future as the surveillance system is developed.

APP also presents a potential economic burden to the country, particularly from the death of individuals at their most economically productive ages. A study conducted in South Korea reports total economic cost of APP equivalent to US$ 150,000,000 which was 0.3% of the cost of total disease [38]. Though not modeled for under-reporting in this study, a recent Tanzanian study [18] reported that mortality from APP in Tanzania was 2.2 cases per 1,000,000. Further research into the economic burden posed by pesticides, both fatal and non-fatal APP, including the impact on health services which have to cope with the burden entirely preventable illness, is warranted.

Despite the focus on acute pesticide poisoning in this study, there is evidence for an association of APP with long term health effects. These include studies showing occupational groups that agricultural workers previously poisoned by cholinesterase-inhibiting pesticides are at risk for chronic adverse effects on the central and peripheral nervous systems. These include farmers in Spain [39], agricultural workers in the US [40], and agricultural workers in Nicaragua [41]. In contrast, short-term exposures in the absence of acute poisoning appear to be unlikely to lead to long-term neurobehavioural deficits [42, 43]. This means that a proportion of the reported individuals suffering APP may be likely to suffer long term sequelae, limiting their productivity and increasing their need for care and that a focus on following up APP cases rather than all persons affected by short-term exposures is warranted for the prevention of long-term effects as well.

There are few limitations to be considered in this study. The study is based on a number of assumptions related to adjusting in the model for missing data and for under-reporting. It is possible that the primary studies on which these extrapolations are based may not have generated valid estimates for modeling. However, the hospital-based reporting showed significant improvements with prospective data collection over retrospective review with respect to completeness and accuracy [24], which suggests reasonable face validity. The farmer survey [20] was, however, based on a relative small sample that was not randomly selected and cannot be regarded as representative of all farmers in Tanzania. However, the age, gender and socio-economic profile of participants was typical of the agricultural workforce in Tanzania. Moreover, the prevalence of poisoning reported by Tanzanian farmers was consistent with a similar study conducted in Central America [34] and the level of under-reporting has also been found to be similar in South Africa [5]. For these reasons, the study findings were thought to be reasonable suitable for use in this modeling. Nonetheless, to account for potential variability in the metrics, extensive sensitivity analyses were used, both for the level of under-reporting and for the attribution of circumstance to poisonings lacking data on circumstance of poisoning. The modeling thus provides very wide estimates which should account for potential variability. Bearing in mind these caveats, we believe the study has generated useful data to reconsider the profile and extent of APP in Tanzania.

## Conclusions

The study confirms that data on APP in Tanzanian hospitals are poorly reported and that occupational circumstances are particularly overlooked in routine facility-based surveillance. Cases involving APP from workplace factors present in routine facility-based surveillance in Tanzania are likely to be just a fraction of the underlying estimates. While the exact extent of under-reporting of occupational poisoning has wide variation in the estimates, depending on the assumptions of the model, it remains clear that APP due to occupational circumstance needs to be taken more seriously in addressing prevention measures. This pattern is consistent with findings in other LMICs where occupational circumstances are often overlooked. A comprehensive surveillance system for APP should consider multiple data sources including community self-reporting in order to achieve better coverage of the at risk population.

## Abbreviations

APP: Acute pesticide poisoning
CI: Confidence interval
FAO: Food and Agriculture Organization
HMIS: Health management information system
IR: Incidence rate
LMIC: Low and medium income countries
MAFS: Ministry of agriculture and food security
NGO: Non-Governmental Organization
PIC: Prior Informed consent
PPE: Personal protective equipment
TAPOHE: Tanzania Association of Public Occupational and Environmental Health Experts
TPRI: Tropical Pesticides Research Institute
WAHSA: Work and Health in Southern Africa
